# Using Jazz as a Metaphor to Teach Improvisational Communication Skills

**DOI:** 10.3390/healthcare5030041

**Published:** 2017-08-04

**Authors:** Paul Haidet, Jodi Jarecke, Chengwu Yang, Cayla R. Teal, Richard L. Street, Heather Stuckey

**Affiliations:** 1 Medicine, Humanities, and Public Health Sciences, Woodward Center for Excellence in Health Sciences Education, The Pennsylvania State University College of Medicine, 500 University Drive (H176), Hershey, PA 17033, USA; 2 Woodward Center for Excellence in Health Sciences Education, Pennsylvania State University College of Medicine, Hershey, PA 17033, USA; jodi.jarecke@gmail.com; 3 Public Health Sciences, Pennsylvania State University College of Medicine, Hershey, PA 17033, USA; chengwu.yang@gmail.com; 4 Clinical and Translational Medicine, Academic Affairs, Texas A&M University College of Medicine, TX 78665, USA; teal@medicine.tamhsc.edu; 5 Medicine, Baylor College of Medicine, Communications, Texas A&M University, College Station, TX 77843, USA; r-street@tamu.edu; 6 Medicine and Public Health Sciences, Pennsylvania State University College of Medicine, Hershey, PA 17033, USA; hstuckey@pennstatehealth.psu.edu

**Keywords:** physician-patient relations, patient-centered care, communication skills, arts and medicine, improvisation, education, medical, music and medicine, professionalism, patient experience, relationship-centered care

## Abstract

Metaphor helps humans understand complex concepts by “mapping” them onto accessible concepts. The purpose of this study was to investigate the effects of using jazz as a metaphor to teach senior medical students improvisational communication skills, and to understand student learning experiences. The authors designed a month-long course that used jazz to teach improvisational communication. A sample of fourth-year medical students (*N* = 30) completed the course between 2011 and 2014. Evaluation consisted of quantitative and qualitative data collected pre- and post-course, with comparison to a concurrent control group on some measures. Measures included: (a) Student self-reports of knowledge and ability performing communicative tasks; (b) blinded standardized patient assessment of students’ adaptability and quality of listening; and (c) qualitative course evaluation data and open-ended interviews with course students. Compared to control students, course students demonstrated statistically significant and educationally meaningful gains in adaptability and listening behaviors. Students’ course experiences suggested that the jazz components led to high engagement and creativity, and provided a model to guide application of improvisational concepts to their own communication behaviors. Metaphor proved to be a powerful tool in this study, partly through enabling increased reflection and decreased resistance to behaviors that, on the surface, tended to run counter to generally accepted norms. The use of jazz as a metaphor to teach improvisational communication warrants further refinement and investigation.

## 1. Introduction

Conceptual metaphor is a linguistic device that helps humans understand and communicate complex concepts by mapping them on to well-known or concrete concepts [1]. Metaphor is powerful, because it forms a bridge between the abstract and the concrete, using images and ideas that are culturally accessible. For the past several years, we have been exploring connections between jazz performance and patient–physician encounters [2], using jazz as a metaphor to explore the improvisational aspects of medical communication. These explorations led us to develop an elective course for fourth-year medical students aimed at fostering students’ improvisational medical communication skills. In this study, we sought to investigate the effects of using jazz to teach communication skills, and to understand the learning processes that students experienced.

## 2. Conceptual Model

**Figure 1:**
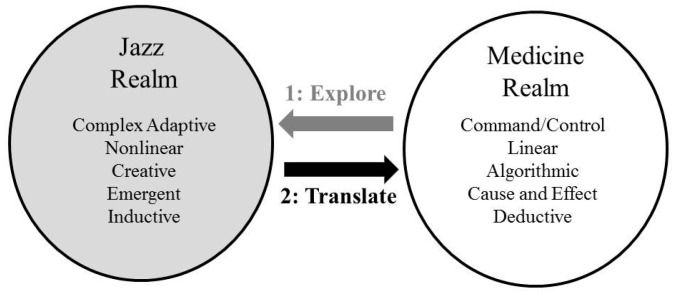
A conceptual model for using jazz to teach communications skills.

The conceptual model for our course (Figure 1) is based on frameworks that use the arts to teach various topics in medical education [3,4,5,6]. We selected jazz as the art because of its focus on improvisation. While many fields discuss improvisation as a central concept, the improvisational part of jazz is well aligned with human conversation. As Ingrid Monson (building on the work of Paul Berliner) has noted, jazz musicians often describe “jazz as a musical language, improvisation as musical conversation, and good improvisation as talking or ‘saying something’” [7,8]. Figure 1 indicates some characteristics of communication in the realms of jazz and medicine. Our strategy in each of the course sessions was to “pull” students from the realm of medicine into the realm of jazz (through guided listening and reflection exercises), thus exploring course communication concepts within the realm of jazz as a first step. The second step was to engage learners in exercises designed to help them to translate their understandings of these concepts back into the medicine realm in a way that they would find relevant, meaningful, and useful to their medical practice. Through repeating cycles of this process, the course itself became improvisational, with teacher and learners engaging in a series of unfolding conversations characterized by back and forth sharing of meaning, insight, and discovery [9]. For the purposes of our course, we defined learning as a substantive change in behaviors or attitudes, measured before and after the course, that would relate to the patterns of communication by course participants.

We made several assumptions about our learners. First, we assumed that many of our learners would have had very little exposure to jazz. Second, since our population of learners consisted of fourth-year medical students, we assumed that, at the outset of the course, they would already have established their own medical communication habits, and might resist adopting nuanced and advanced levels of skill in domains where they already felt competent. We therefore designed our activities to use the jazz metaphor to foster student exploration of course concepts in unfamiliar (i.e., jazz music) conceptual territory. We hypothesized that, since many of the students would not have had substantive exposure to jazz prior to the course, the process of immersing first in jazz would help to minimize preconceived notions about communication that might act as barriers to students adopting new behaviors. In addition, we wanted to expose students to situations that are nonlinear and emergent, requiring listening, inductive thinking, and complex adaptive decision making [10]. While these characteristics could describe medicine as well as jazz [11,12,13], much of the literature on medicine’s culture speaks to the contrary. This literature suggests that, by the fourth year, students have been acculturated into a hierarchical environment wherein “command and control” decision-making is the norm, and many adopt the belief that medicine is characterized by linear, cause-and-effect problems best solved only by algorithmic and deductive thinking [14]. Assuming that at least some of our students would espouse such beliefs, we purposely designed our course to guide students through repeated cycles of first exploring particular communication concepts within the metaphor of jazz, followed by guided activities to translate each concept back into medicine.

## 3. Course Design

The “Jazz and the Art of Medicine” course is four weeks in duration, and includes 12 h of in-class or simulation activities (3 h each week), and 8 h of clinical practice. In addition, students complete one 90-min writing assignment per week. Each week of the course is devoted to one improvisational communication topic. The topics include:

- Balancing communicative structure with communicative freedom when talking with patients
- Listening for deep meanings in patients’ communications
- Developing one’s own authentic “voice” as a communicator [15]
- Effectively using space (including communicative, physical, psychological, and topical) in the medical encounter [16]

A detailed example of the third session (developing one’s “voice”) appears in Appendix A. All course teaching was done by one of the authors (PH), who has background in medical education, communication skills training, and jazz, specifically consisting of work as a physician and patient–physician communication researcher, jazz radio station disc jockey and program director (WPSU FM 91.5, 1985–1987), and as a current member of the board of directors of the Central Pennsylvania Friends of Jazz (www.friendsofjazz.org). During the weekly class sessions, students first participated in a series of guided jazz music listening exercises and discussions. Our selection of jazz pieces for the course was mainly driven by their salience for discovery about the topic of the session. For example, during the “voice” session, as demonstrated in Appendix A, we chose different versions of the same song by different artists to foster learner exploration of how the conversation and the meaning of what is being said is influenced by the persona of the conversational participants. In this particular example, comparing and contrasting singers Sarah Vaughn and Billie Holiday, and pianists Ahmad Jamal and Bill Evans served this purpose well. For the entire course, we used a variety of selections spanning traditional jazz to jazz fusion.

After exploring concepts within the jazz realm, students translated insights and ideas about each communication concept from jazz to medical practice, using a trigger video of a medical interview and a series of questions for reflection and discussion. After each weekly class session, students spent 2–3 h participating in the care of patients in order to have an opportunity to apply the new communication concept. We secured placements in outpatient clinics in the specialty that each student intended to pursue, and instructed students to practice the concept and explore how it applied or could be applied within their chosen specialty. We instructed clinical preceptors in these settings to assign students to provide direct supervised patient care, so that students would experience the flow and pace of the typical work environment, while also applying the communication concepts [17]. Finally, we gave students a weekly reflective writing assignment that synthesized the multiple learning experiences (jazz, medical translation, clinical practice) into a plan for ongoing communicative practice and personal development.

In addition to the classroom and clinical activities, each student interviewed a standardized patient (SP) pre- and post-course. We audio recorded the SP encounter at each time point, provided students with their own recordings, and prompted students to review the recording from the pre-course session as they worked on their weekly reflective writing assignments. The case we used for the standardized patient has been previously described [18], and presents an advanced cross-cultural communication challenge, with the actor portraying communicative clues that signify important contextual history. She divulges information only if the student recognizes and specifically explores the clues. This case, similar to others that have been described [19], is based on the notion that practicing physicians often commit contextual errors by missing key patient-centered information, ignoring patient clues, and using a high control style during the medical encounter [20,21,22,23,24]. Since the improvisational concepts we taught were aimed at fostering communicative adaptability and advanced listening abilities, we hypothesized that, if the course were successful, students would improve their performance from the first to the second time point.

## 4. Evaluation Design

All aspects of our evaluation design were approved by the Penn State College of Medicine Institutional Review Board. We focused our evaluation strategy on changes in students’ knowledge, attitudes, and behaviors, and collected three types of data related to these learning outcomes. First, we distributed a survey to students at the beginning and end of the course that included student self-assessments of knowledge related to communication skills and ability in performing tasks related to the overall objectives of the course (see Appendix B). The survey also included the Patient Practitioner Orientation Scale (PPOS) [25], the Mindful Attention Awareness Scale (MAAS) [26], and self-ratings of communication confidence based on items from the Harvard Medical School Communication Skills Form [27]. These scales measure attitudes toward patient-centered care (PPOS), mindful practice (MAAS), and communicative tasks (Harvard Communication Skills Form) related to essential communication elements described in the Kalamazoo Consensus Statement [28].

Second, we measured patient-perceived communication outcomes based on each student’s behaviors with the standardized patient at the beginning and end of the course. For each student, the SP completed a survey immediately after the interview that included measures of: (a) the degree to which she felt listened to; and (b) the degree of adaptability which the student demonstrated toward her communication and narrative during the medical interview. The adaptability items appear in Appendix B and the listening items have been previously published [29].

For the standardized patient portion of our evaluation, we also recruited a control group consisting of 10 fourth-year medical students with backgrounds similar to those that completed the month-long course. We informed the control group that they were participating in a study of medical student communication, and asked them to interview the SP twice (an initial interview and a second time one month later) under identical conditions to those of the course students. We audio recorded the interviews and made these recordings available to control students in the month between their two interviews, but provided no further instruction. The standardized patient completed the same survey immediately after control students’ interviews. We did not inform the SP about the course or control status of the students.

We compared quantitative student survey data at the beginning and end of the course using the Wilcoxon signed-rank test because the data were not normally distributed and the sample size was small. For the standardized patient data, we compared mean scores between course and control students at each time point (baseline and one-month) using two-sample *t*-tests when the data were normally distributed and the Wilcoxon–Mann–Whitney test when the data were not normally distributed. Similarly, to compare students’ performance across the two time points, we used either paired *t*-tests or the Wilcoxon signed-rank test. We used Cohen’s D to assess effect sizes.

Finally, since the course was part of a humanities selective graduation requirement for Penn State students, all students completed a standardized course evaluation administered by the Penn State Department of Humanities. We collected qualitative comments from these evaluations, and augmented these data with one-hour individual semi-structured interviews with six of the eight students in the 2011 cohort. Interviews were conducted by an educational researcher (JJ), who was not directly involved in teaching course sessions, and who had not had prior contact with students. In an effort to understand learners’ perspectives, the interviews probed experiences during the various components of the course, and effects of various course activities on perceptions and motivation regarding medical communication. We approached the qualitative data by performing an analysis of student qualitative evaluation comments and transcripts of student interviews through close reading and discussion between two of the investigators (PH and JJ). These investigators used a narrative framework to approach the data, focusing on learner stories and their meanings through an analysis of storied elements such as character, setting, plot, and agency [30]. This analytic dyad was balanced by participation of the course teacher (PH) and an independent educational researcher (JJ). Both were careful to examine their own assumptions as the analysis unfolded. In an effort to check the conclusions drawn, a third investigator not involved in teaching or collection of data, but who is versed in qualitative analysis (HS) reviewed the data, codes, and conclusions to corroborate the content.

## 5. Results

Thirty fourth-year students in four yearly cohorts (2011, eight students; 2012, seven students; 2013, six students; and 2014, nine students) completed the course. Sixteen students were female. The specialties that students planned to pursue included anesthesia (two students), emergency medicine (one student), family and community medicine (four students), internal medicine (five students), neurology (two students), obstetrics and gynecology (five students), otolaryngology (one student), pathology (one student), pediatrics (three students), psychiatry (one student), radiology (two students), and surgery (three students). Ten fourth-year students participated in the control group and completed the two standardized patient interviews; six of these students were female. Specialties that control students planned to pursue included dermatology (one student), emergency medicine (two students), internal medicine (one student), obstetrics and gynecology (two students), pediatrics (three students) and surgery (one student). We performed preliminary analyses on the data from the first eight course students and ten control students in 2011. Since the results of those analyses did not differ substantively from those for the entire cohort, we report results from combined data across all four years of course students.

Results of the course student survey appear in Table 1. As shown in the table, student self-assessments of knowledge improved on all four global knowledge items. In addition, student self-assessments improved on a composite rating of seven abilities related to the objectives of the course. Student attitudes toward patient-centered care and mindful practice did not change over the period of the course. Finally, students’ ratings of confidence in completing essential communication tasks improved over the period of the course.

**Figure 2:**
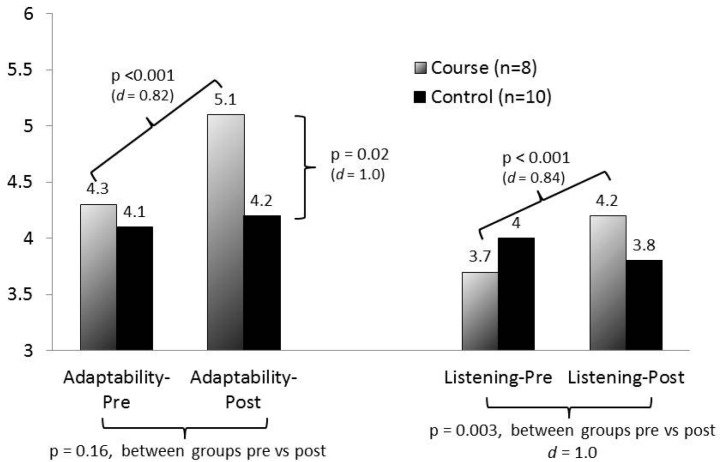
Standardized patient evaluations.

Standardized patient outcomes appear in Figure 2. All statistically significant outcomes are indicated in the figure. The course group demonstrated significant gains from pre- to post-course in both adaptability and quality of listening. While there were no significant differences between the course and control groups on the pre-course measures, the course group scored higher on the post-course adaptability evaluation, and gained significantly more than the control group on the listening evaluation. Cohen’s D scores indicated large effect sizes (d > 0.8) for all statistically significant comparisons.

We focused our qualitative data collection and analyses on probing students’ course experiences, specifically in relation to our conceptual model. Based on our analysis, four important themes emerged. First, students described the course as an engaging classroom experience, wherein they were actively involved and invested in the course content and activities:

- “We were able to incorporate the musical concepts with the patient concepts, and so it made the time go by faster. It kept us engaged the entire time. I thought that was the strongest point [of the course].”
- “So it kept us engaged. A lot of times, time flew by…we didn’t even realize when the time was over because we were all having fun…It was very, very interactive. It got you thinking.”
- “I never thought you could use music to learn about communication. And here, [the instructor]…not only taught me about music but also taught me about communication…He made it fun and he made it not rigid…I thought it was a great way to do it.”

Second, students indicated that, in contrast to previous didactic classroom experiences focused on communication, the use of jazz provided a fresh approach to learning, facilitating new and creative ways to communicate with patients:

- “Because I'm not [familiar with] jazz, I had to think differently from the beginning. I had to think outside the box. My brain was being used in ways I wasn’t used to, and that made it easier to learn concepts about communication, whereas if this was in the standard classroom, no music, no talking, and even [just] a standardized patient, I don’t think I would have been as open and ready to try new things as much as I was.”
- “…for 3 ½ years we're taught a very structured technique of talking to patients. And so to do something different…to communicate it in a different way has been interesting.”
- “I definitely gained a whole new perspective on the music of jazz and also I think the art of communication…there were similarities and things that we could learn from the music and then about ourselves and what we were doing as far as our communication skills.”

Third, in addition to helping students to approach communication differently, participants suggested that the jazz metaphor also provided a model to guide their understanding of communication concepts:

- “I think it’s one thing to just be told that this is what you are supposed to do, but another thing to hear the music and see these musicians who are doing the same thing in their form of communication and to be able to use that as a model for us in terms of the communication that we need with the work we do.”
- “By using jazz, it was a great model to…help us understand communication in a way that is relevant and in a way that almost all of us can relate to.”
- “it probably makes the concept stick a little bit better because you have a visual, or an audio in this case….even if you forget the concepts, you can always think back to that and remember, ‘Oh yeah, in jazz they do this…’”

Finally, students suggested that they became increasingly aware of their own agendas as they interacted with patients. A key recognition was that communication checklists taught in various history-taking courses often dominate their interviews and leave little space for patients to tell their stories:

- “I summarized the course for myself and said, ‘step away from your notes and your list of questions and your list of fill-in-the-blanks and just have a conversation.’”
- “Being a fourth-year, you think you know it all at this stage and by the time we went through the course, I was like: ‘I know nothing about communication’…I need to revamp the way I talk to patients and how I gather information from patients”
- “It takes more of a mental effort to sort out what the patient is saying. It also means that you're not really in control anymore, the patient is in control. And that shift mentally for a medical student or for a doctor is pretty—it’s challenging because you want to be in control. You want to be the doctor. But, to communicate effectively…it’s not the right thing to do.”

## 6. Discussion

Why use the arts, and jazz in particular, to teach medical communication skills? After all, the communications literature is well populated with topical frameworks [28,31,32,33] and proven methods to promote better communication skills [34,35,36]. The uniqueness of this study lies not necessarily in the actual skills that students practiced. Rather, the major innovation of our course lies in using jazz to provide a metaphorical frame for student discovery and understanding of improvisational communication processes. While we were pleased that students in this course demonstrated self-reported gains in knowledge and communicative skills as well as substantive improvements in standardized patient-assessed performance compared to controls, we believe that our qualitative data provide clues to two important events that represent a new direction for communications training programs.

First, students repeatedly talked about having lowered resistance to trying out new communicative strategies and adjusting previously used strategies. In order to develop narrative competence [37], physicians need to develop a skill set that often runs counter to generally accepted ways of conducting the interview. Such skills include the ability to collect information in a non-linear fashion, and to share control with the patient over communicative processes, in effect “co-constructing” a history with the patient rather than “taking” it from them [38]. However, many students and practicing physicians may have an inclination to dismiss such notions as unrealistic under the pace and time pressures of real-world practice. The value of spending time exploring such communication behaviors in the jazz realm lies in jazz’s foreignness compared to medical practice. Since most students were not very familiar with jazz at the outset of the course, they did not have many preconceived notions about what is and is not realistic within jazz, so were able to take the course’s communication concepts more at face value than immediately dismissing them without due consideration. By first starting class sessions with discussions about jazz, students may have been able to develop a different understanding of each of the four communication concepts of the course, and this different understanding may have primed them to more seriously explore how each concept would operate within their own specialty and medicine in general.

Second, we propose that the central jazz theme of improvisation provided an overall umbrella to guide integrating multiple individual communicative acts into students’ ongoing behaviors. For example, during the session on communicative space, students explored several distinct communicative skills, including using silence, pacing, communicative latencies (the time between turns at talk), and open-ended questioning, all aligned toward having a good improvisation process with patients. This idea of “good improvisation” was clarified by the jazz listening exercises, and provided a concrete frame in which to explore and practice individual communicative skills, such as asking open-ended questions. When students subsequently attended clinical sessions as part of the course, they did so not only with specific behaviors to try, but also with a vision of the kind of harmonious improvisation that those behaviors were intended to create, and they were prepared to experiment and start figuring out for themselves how such behaviors would fit together in achieving this aim.

We believe this study suggests a new way of thinking for medical educators who have often approached teaching communication to future physicians with lists of best practices, key phrases to memorize and use, and various questioning techniques. While using the arts to teach medical topics is not new, this study suggests that medical students may respond positively to jazz concepts as a way to understand and use, in practice, communicative presence, adaptability, and engagement with patients, particularly in response to intentional pedagogical strategies employed to maximize the learning both within the jazz and medical realms. As promising as our results are, however, this study raises many questions about implementation that may influence the subsequent learning impact, and these need further study. For example, it is unlikely that students automatically make connections between the art and their own medical practice; how can these connections be enhanced by the educator? What pedagogies and strategies can teachers use to maximize the effectiveness of translation from the art to the bedside? We have proposed a set of strategies in our curriculum, but other approaches may be equally or more effective; what are such techniques, and can they engage students who have no or only a passing interest in jazz? Can the lessons learned in this study be broadly applied to the use of the arts in general education [6]?

Our study has several limitations. First, even though we employed a control group for the standardized patient evaluation, it represents a single study at a single school. Penn State is the first medical school in the US to establish a Department of Humanities and has a reputation for teaching Medical Humanities. Its students may therefore be somewhat unique, and many cite the Humanities presence as a factor in choosing to attend this school. It would be illuminating to study the course at additional schools. Second, the outcomes we studied represent immediate changes in behaviors and attitudes, and may not reflect long-term changes in the participants’ medical practice. Additional study is needed to assess the downstream effects of this intervention.

## 7. Conclusions

In conclusion, our experience with this course suggests that using jazz as a metaphor can be a powerful tool in fostering patient-centered communication. This power is derived partly from an ability to suspend resistance to behaviors that may run counter to generally accepted norms [39,40]. We believe that the use of art as metaphor in general, and jazz to teach improvisational communication in particular, warrants further refinement and investigation.

## Figures and Tables

**Table 1:** Student Survey Results *.

Evaluation Item or Instrument **	Pre-Course {Median, [95% CI]}	Post-Course {Median, [95% CI]}	*p* Value
Self-Rated Knowledge Items			
Knowledge of Jazz	2 [2, 3]	4 [4, 5]	<0.001
Enjoyment of Jazz	4 [4, 5]	5 [4, 6]	0.002
Understanding of Improvisation	4 [3, 4]	5 [5, 6]	<0.001
Understanding of Patient–Physician Communication	5 [5, 6]	6 [6, 6]	0.003
Self-Rated Ability to Perform Tasks Related to Course Objectives	5.2 [5.0, 5.5]	5.5 [5.2, 5.7]	0.005
PPOS Score *** (6-point scale)	4.3 [4.2, 4.5]	4.3 [4.2, 4.6]	0.6
MAAS Score *** (6-point scale)	3.9 [3.7, 4.1]	3.8 [3.4, 4.1]	0.2
Communication Confidence Score *** (6-point scale)	4.0 [3.9, 4.1]	4.3 [4.1, 4.4]	0.01

* Comparison of student knowledge and attitudinal self-assessments before and after the course; ** All surveys used a seven-point scale except where otherwise noted; *** PPOS = Patient–Practitioner Orientation Scale, MAAS = Mindful Attention Awareness Scale, Communication Confidence Score items adapted from the Harvard Communications Skills Form.

## Previous Presentations

Earlier versions of this work were presented at the International Conference on Communication in Healthcare, St Andrews, Scotland, September 2012, and the Annual Meeting of the Association of American Medical Colleges, San Francisco, California, USA, November 2012.

## Appendix A. Example of Class Session: Voice

### *Session* *3: Finding/Developing Your Voice (Teaching Outline)*

#### A. Introduction

In this session, we will explore elements of “voice”. This session is a bit abstract, and while people often use the word “style” interchangeably with “voice”, style is only one aspect of one’s voice. Without specifically defining voice, the session is designed move through some listening and viewing exercises aimed at thinking about various aspects of voice, allowing learners to formulate their own conceptual definition of the concept.

The objectives for the session are as follows: By the end of this session, learners should be able to:

1) Articulate key communication elements that lead to an impression of style;
2) Understand how others articulate elements, and keep or modify one’s own impressions as a result;
3) Articulate one’s own internal preferences and worldview that shapes their voice;
4) Plan to try out new communication strategies.

Without specifically defining voice, here is a bit of background information:

If one wants to become a jazz musician, they must first learn the fundamentals. They must have a comprehensive working knowledge of all the scales (e.g., c major, a minor, b-flat major, etc., etc.), and all the songs (called “jazz standards”—there are anywhere between 250 and 500 of them) that are played in jazz. They must be able to play any song in any key at the drop of a hat. They must have complete mastery of their instrument; they must know their instrument so well that they can just think of a phrase and their fingers will automatically play it without them having to think about the fingering, where to pluck the string, how hard to blow, etc.

However, if this is all one does, they will never be a great jazz musician. There are plenty of musicians who have mastery of their instruments. There are even some who know all of the jazz scales and songs. The truly good and great jazz musicians have all gone one step further—they have developed their VOICE on their instrument. This is more than style; I think of it as more like channeling their own personal vision, conception, ideas, and personhood through their instrument. A serious jazz fan can tell the difference between John Coltrane and Sonny Rollins (two great tenor saxophonists in the 50 s and 60 s) within 3 notes, and can identify them amid the hundreds of saxophonists who played in that era. That’s because Coltrane and Rollins had fully developed and distinctive voices.

#### B. Jazz listening exercises

First Exercise: Play for the students two versions of the song “They Can’t Take That Away From Me” by Sarah Vaughn and Billie Holiday. (Sarah Vaughn from the 1957 album “Swingin”, and Billie Holiday from the 1957 album “Songs for Distingue Lovers”) These two versions were released in the same year, are done in the same key, and are played at the same speed. Also provide students with a sheet with the lyrics to the song. Have students take a quick look at the lyrics, then listen first to the Sarah Vaughn version, and next to the Billie Holiday version.

Have students discuss the following prompts in groups of 2–4:

1) List 3 defining characteristics of each singer’s “style” while you listen. How do those elements of style relate to what you interpret the song to be about?
2) Now, discuss briefly what you interpret the biggest DIFFERENCE and SIMILARITY between the two singers to be. In other words, what is different between them? What is similar?
3) The final question is the most abstract, but most important question: Which one of these singers would you most like to “be like” as a physician? Spend some time pondering this one, and try to think “out of the box” about this.

After the students have discussed the prompts, have the groups write their answer to the final prompt (Vaughn or Holiday) on a sheet of cardstock, then hold it up, so that all groups can see which singer the other groups picked. Facilitate a discussion among all of the groups about what led them to choose the singer that they chose.

Second Exercise: Repeat the same process, this time using two piano players. Since there are no words, this means that students will have to listen to and derive meaning from the other aspects of language, such as the sounds, arrangements of notes, speed, or whatever aspects of the music that their minds gravitate toward. (This is analogous to the nonverbal and paraverbal (i.e., the sounds) language used between doctors and patients).

Play for the students two versions of the song “Emily”, originally written by Johnny Mandel and Johnny Mercer. (The first version is by Ahmad Jamal from the 1968 album “Tranquility”, and the second is by Bill Evans from the 1967 album “California Here I Come”.) Invite students to focus specifically on the musical “voice” of each pianist as they listen to the solos during each track.

As with Sarah Vaughn and Billie Holiday, have students discuss the following prompts in small groups of 2–4, followed by a large group discussion of the second prompt:

1) List 3 defining characteristics of each instrumentalist’s “voice” as you listen. Based on what you hear, speculate on what you think these three musicians “are like” as people. If you met them on the street, what would your first impressions of them be? Why? What did you hear that led you to these speculations?
2) Now, once again: Which one of these instrumentalists would you most like to “be like” as a physician? Spend some time pondering this one, and try to think “out of the box” about this. Be prepared to describe why you chose the instrumentalist that you did.

#### C. Patient-Physician Communication Exercise

Show the students a video of a doctor-patient encounter (The video we use can be provided by contacting Paul Haidet at phaidet@pennstatehealth.psu.edu).

Direct students to pay particular attention to the doctor’s voice as they watch.

Discussion prompts (students discuss in groups of 2–4, followed by facilitated classroom discussion):

1) In 3 words or less, describe this doctor’s voice. Explain what language or communicative behaviors you saw and heard that led you to choose the words you did.
2) Would you consider this doctor to be a “patient centered” doctor? Why or why not? What did you see and hear that shaped your impressions?
3) Which of the two singers was this doctor’s voice most similar to? Explain your choice.
4) Which of the three instrumentalists was this doctor’s voice most similar to? Explain your choice.

This completes the in-classroom portion for week #3. After the in-class session, students attend a 4-hour clinic session (within their chosen specialty), working to take care of patients. Within two days of completing the clinic session, each student should complete and submit a writing assignment in response to the following prompts:

#### D. Writing Prompts (post-clinic)

As you write, try to keep focused on your core being (e.g., who ARE you?), and the communicative elements that go with that core being:

1) Describe your “voice.” What are its distinguishing characteristics right now, and what distinguishing characteristics do you want to develop as you move forward in your career?
2) What three words would you want patients to use to describe YOUR voice?
3) How will you know if your patients will perceive you that way?
4) Extra Credit: With what types of patient “voices” will you have difficulty maintaining your own voice? Why?

## Appendix B. Evaluation Items

### *A.* *Pre- and Post-Course Student Survey Items*

- Knowledge items (7-point response choice for each item, anchors applied to the lowest and highest response choices)
  i. Rate your knowledge of jazz (“little or no knowledge”—“comprehensive knowledge”)
  ii. Rate your enjoyment of jazz (“little enjoyment”—“enjoy jazz a great deal”)
  iii. Rate your understanding of improvisation (“little or no understanding”—“comprehensive understanding”)
  iv. Rate your understanding of patient-physician communication processes (“little or no understanding”—“comprehensive understanding”)
- Self-assessment of abilities related to course objectives (students instructed to rate their level of confidence in being able to do each behavior right now using a 7-point response scale anchored by “not confident at all” and “completely confident”)
  i. Adapting when interacting with patients
  ii. Using my own personal style when interacting with patients
  iii. Giving patients space to talk while also managing time effectively
  iv. Paraphrasing what I have heard patients say in a way that feels natural and unforced
  v. Understanding patients’ unique perspectives
  vi. Being perceived by patients as a “good listener”

### *B.* *Standardized Patient Adaptability Assessment Items (6-point Likert response scale for each item)*

- The student modified his/her pace of speech to be more similar to mine.
- The student modified his/her tone to be more similar to mine.
- When I offered my beliefs about my symptoms, the student responded in a non-judgmental way.
- When I verbally indicated uncertainty/confusion, the student clarified or explained in language I could understand.
- When I non-verbally indicated uncertainty/confusion, the student followed up with a question or probe.
- This student was able to understand the impact of my life circumstances on my illness.
- Overall, this student was skilled at recognizing my non-verbal cues (body language, facial expressions, eye contact, fidgeting).
- Overall, this student was skilled at recognizing my verbal cues (word choice, tone of voice, use of pauses and emphasis).
- Generally, during the encounter the student seemed open to my input.
- Generally, during the encounter the student was able to include my input in our discussion.
